# Frequency and phenotype of B cell subpopulations in young and aged HIV-1 infected patients receiving ART

**DOI:** 10.1186/s12977-014-0076-x

**Published:** 2014-09-11

**Authors:** Sylvie Amu, Gitit Lavy-Shahaf, Alberto Cagigi, Bo Hejdeman, Silvia Nozza, Lucia Lopalco, Ramit Mehr, Francesca Chiodi

**Affiliations:** Department of Microbiology, Tumor and Cell Biology, Karolinska Institutet, Nobels väg 16, Stockholm, 17177 Sweden; The Mina and Everard Goodman Faculty of Life Sciences, Bar-Ilan University Ramat-Gan, Ramat-Gan, Israel; Department of Infectious Diseases, Stockholm South General Hospital, Stockholm, Sweden; Department of Infectious and Tropical Diseases, San Raffaele Scientific Institute, Milan, Italy; Division of Immunology, Transplantation and Infectious Diseases, San Raffaele Scientific Institute, Milan, Italy

**Keywords:** HIV-1, B cells, Aging, CD25, CD69, IL-6

## Abstract

**Background:**

Aged individuals respond poorly to vaccination and have a higher risk of contracting infections in comparison to younger individuals; whether age impacts on the composition and function of B cell subpopulations relevant for immune responses is still controversial. It is also not known whether increased age during HIV-1 infection further synergizes with the virus to alter B cell subpopulations. In view of the increased number of HIV-1 infected patients living to high age as a result of anti-retroviral treatment this is an important issue to clarify.

**Results:**

In this work we evaluated the distribution of B cell subpopulations in young and aged healthy individuals and HIV-1 infected patients, treated and naïve to treatment. B cell populations were characterized for the expression of inhibitory molecules (PD-1 and FcRL4) and activation markers (CD25 and CD69); the capacity of B cells to respond to activation signals through up-regulation of IL-6 expression was also evaluated. Increased frequencies of activated and tissue-like memory B cells occurring during HIV-1 infection are corrected by prolonged ART therapy. Our findings also reveal that, in spite of prolonged treatment, resting memory B cells in both young and aged HIV-1 infected patients are reduced in number, and all memory B cell subsets show a low level of expression of the activation marker CD25.

**Conclusions:**

The results of our study show that resting memory B cells in ART-treated young and aged HIV-1 infected patients are reduced in number and memory B cell subsets exhibit low expression of the activation marker CD25. Aging per se in the HIV-1 infected population does not worsen impairments initiated by HIV-1 in the memory B cell populations of young individuals.

**Electronic supplementary material:**

The online version of this article (doi:10.1186/s12977-014-0076-x) contains supplementary material, which is available to authorized users.

## Background

The administration of highly active antiretroviral therapy (ART) to HIV-1 infected patients has led to improved health conditions and increased life expectancy in many treated individuals. As an example, it has been reported that in the United States by 2015, half of the ART treated patients will reach age 50 or older [1]. This increase in life expectancy and age does not always relate to a life free of illness conditions: in fact non-AIDS conditions, including cardiovascular diseases, osteoporosis, renal and liver diseases, neurocognitive impairments and cancer, are all increasing in this group of HIV-1 treated patients [1].

A tremendous variety of immune dysfunctions that takes place during HIV-1 infection can be directly linked to the replication of the virus in target cells but also to bystander mechanisms of immunological damage triggered by the virus. Microbial translocation through the damaged epithelial barrier in the gut [2] fuels events leading to inflammation and apoptosis of immune cells in lymphoid compartments and circulation. The continuous immunological activation taking place during chronic HIV-1 infection may lead to immunological features which are signatures of aging in healthy individuals. In this respect the term “exhaustion” is often used to characterize poor responses to activation signals by expanded T-cell populations during HIV-1 infection [3].

B cells can be divided into several distinct subpopulations according to lineage and differentiation markers; the characterization of B cell subpopulations in the blood of HIV-1 infected patients has revealed that profound alterations take place in the composition of the B cell pool during HIV-1 infection [4,5]. Immature transitional B cells, which derive from progenitor B cells residing in the bone marrow (BM) characterized by the expression of the CD10 lineage marker and the absence of CD27 expression [6,7], tissue-like memory B cells similar to tonsillar B cells in expression of the inhibitory receptor Fc-receptor-like-4 (FcRL4) [8] and B cells with plasmablast characteristics and low CD21 expression classified as activated memory B cells [9] are all increased in the blood of viremic HIV-1 infected individuals.

In contrast, a reduced number of resting memory B cells has been found in blood samples of HIV-1 infected patients [10-12]. Memory B cell functions improve by early initiation of ART in adults and children [13,14]. However, once the depletion of memory B cells is established during chronic HIV-1 infection, the replenishment of this memory B cell population may be difficult to correct, even following a prolonged period of treatment. Accordingly, the administration of ART during chronic HIV-1 infection leads to normalization of all the B cell subsets with the exception of resting memory B cells [15]. That the proportion of resting memory B cells remains low in spite of viremia control indicates that several pathological mechanisms may impact on B cell dysfunctions during HIV-1 infection, including T-cell lymphopenia and hyperactivation [16].

This persistent feature of B cell immunopathology may have relevant clinical consequences, as resting memory B cells are responsible for serological memory against vaccination antigens and pathogens encountered during life. Accordingly, the reduction of memory B cells during HIV-1 infection may lead to a weakened B cell immunity to vaccines [17-20]. Interestingly, effective humoral immunity is also compromised in healthy individuals during aging, with poor antibody response to many vaccines and increased susceptibility to infections which rely on antibody control [21]; this weakened immune response in elderly people has been linked to impairment of somatic hypermutation and class switch recombination as reviewed in [22,23].

The effect of age on the distribution of B cell subpopulations has not been evaluated in detail [24]; furthermore, it is unknown whether increased age during HIV-1 infection further synergizes with the virus to affect the distribution of B cell subpopulations. In the present work we evaluated the impact of age on the distribution of B cell subpopulations in healthy individuals and HIV-1 infected patients, treated and naïve to treatment. With the purpose of pin-pointing the possible impact of age and HIV-1 infection on the phenotype of B cell subpopulations, we measured the expression of inhibitory molecules (PD-1 and FcRL4) and activation markers (CD25 and CD69) on B cell subpopulations in healthy controls and HIV-1 infected subjects, and related these findings to the ability of B cells to respond to activation signals. A detailed statistical analysis is presented to characterize the impact of age and length of treatment on phenotypical changes occurring in B cell subpopulations during HIV-1 infection.

## Results

### Effect of HIV-1 infection, ART and aging on B cell subpopulations

We initiated the study by assessing the frequency and changes induced by HIV-1 infection in Naive CD19^+^CD10^**−**^CD21^+^CD27^**−**^, Activated Memory (AM) CD19^+^CD10^**−**^CD21^**−**^CD27^+^, Resting Memory (RM) CD19^+^CD10^**−**^CD21^+^CD27^+^ and Tissue Like Memory (TLM) CD19^+^CD10^**−**^CD21^**−**^CD27^**−**^ B cell subsets in the three groups studied (controls, HIV-1 non-treated and HIV-1 treated) independently of age (Additional file 1: Figure S1 and Additional file 2: Figure S2). As previously reported [15], HIV-1 infection leads to a significant reduction of the RM B cell subset that is not corrected by treatment (P < 0.001) when comparing treated and non-treated individuals to controls. Furthermore, HIV-1 infection results in a significant increase in the frequency of AM B cells (P < 0.05 and P < 0.001) when comparing treated and non-treated individuals to controls; this increase was corrected to some extent, but not normalized, by treatment (P < 0.05) (Additional file 2: Figure S2). The significantly increased percentages of TLM B cells (P < 0.001) identified when comparing non-treated and treated individuals to controls were partially corrected by ART administration (P < 0.01) (Additional file 2: Figure S2). We did not identify a significant difference in the frequencies of naive B cells between the groups studied.

In order to investigate the influence of age, and age in combination with HIV-1 infection, on the frequency of peripheral blood B cell subsets, the study subjects were divided into three age categories: 30 years or less (defined as young), between 31 to 50 years (defined as middle) and 51 years and above (defined as aged). Table 1 shows the descriptive statistics of subject age in each age group and population.

**Table 1:** **Clinical, virological and immunological characteristics for individuals included in the study**

**Population with age group**	**nr**	**Age range (mean)**	**CD4 cells/μl (range)**	**CD8 cells/µl (range)**	**CD4:CD8 ratio**	**Virus load copies/ml (range)**	**Length of ART months (range)**
Control < =30	27	21-30 (25.85)	ND	ND	1.00 (0.41-1.84)	NA	NA
Non-treated < =30	14	23-30 (25.36)	628 (394–1096)	1188 (630–2096)	0.61 (0.23-1.19)	102070 (20-1×10^6^)	NA
Treated < =30	18	21-30 (25.61)	580 (308–1169)	862 (307–1567)	0.68 (0.32-1.33)	156 (0–893)	9.2 (1–48)
Control 31 = <<=50	38	31-50 (39.97)	ND	ND	1.93 (0.23-5.47)	NA	NA
Non-treated 31 = <<=50	10	31-47 (38.30)	559 (278–1357)	985 (421–1762)	0.7 (0.19-2.03)	33132 (233–1.3×10^5^)	NA
Treated 31 = <<=50	24	31-50 (41.88)	589 (222–2010)	884 (322–2175)	0.79 (0.15-1.96)	6 (0–36)	74.1 (2–160)
Control > =51	36	51-87 (58.83)	ND	ND	2.12 (0.88-5.17)	NA	NA
Non-treated > =51	11	51-85 (64.36)	604 (336–1235)	1334 (613–3185)	0.51 (0.16-0.92)	39886 (0–1.2×10^5^)	NA
Treated > =51	28	51-76 (57.46)	599 (247–1412)	895 (473–1452)	0.74 (0.20-2.99)	25.2 (0–343)	64.1 (2–163)

ND = Not Done.

NA = Not applicable.

**Figure 1:**
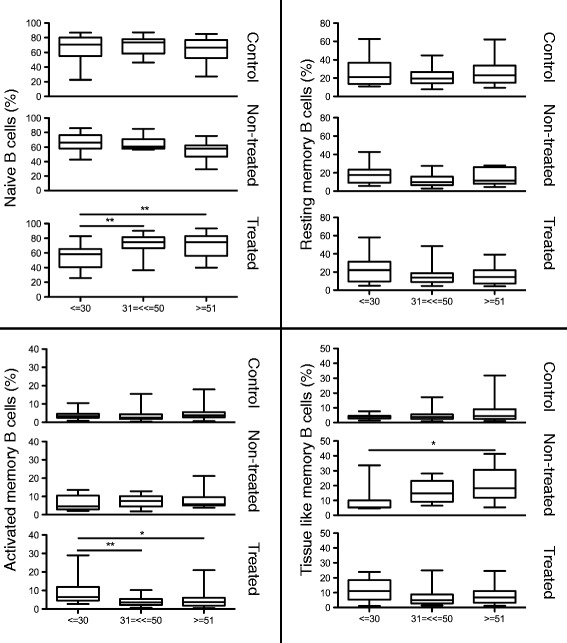
**Percentages of B cell subsets in relation to age groups.** The average percentages of B cell subsets in patients belonging to the age groups < =30, 31–50 and > =51 years of age in healthy controls, non-treated and treated HIV-1 infected patients are shown. The specimens included in the figure were obtained from the subjects presented in Table 1. The results are illustrated for the different subsets: Naive, resting memory (RM), activated memory (AM) and tissue-like memory (TLM) B cells. * = P < 0.05, ** = P < 0.01.

The comparisons of the average percentages for B cell subsets showed significant differences between age groups, mostly in the B cell subsets of treated individuals (Figure 1). The middle and aged treated groups showed significantly increased percentages of naïve B cells (P < 0.01) and significantly decreased levels of AM (P < 0.01 and P < 0.05, respectively) B cells when compared to the young treated group (Figure 1). A significant increase of TLM B cells was seen in the non-treated group when comparing young and aged (p < 0.05). A decline of naive B cells according to age was noticed in non-treated patients (66.04%, 64.41 and 55.86 respectively) although this difference did not reach significant levels.

**Figure 2:**
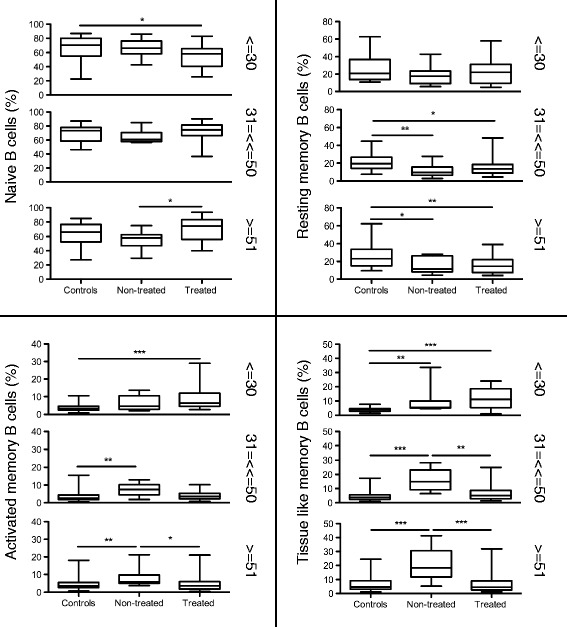
**Percentages of B cell subsets in relation to HIV-1 infection and treatment.** The average percentages of B cell subsets in controls, non-treated and treated HIV-1 patients are shown for each age group (<=30, 31–50 and > =51 years of age). The specimens included in the figure were obtained from the subjects presented in Table 1. The results are illustrated for the different subsets: naive, resting memory (RM), activated memory (AM) and tissue-like memory (TLM) B cells. * = P < 0.05, ** = P < 0.01, *** = P < 0.001.

Furthermore, we assessed the impact of HIV-1 infection and ART on B cell subpopulations for the different age groups (Figure 2). The level of naive B cells significantly increased after ART treatment in the aged group (P < 0.05) when compared to the non-treated group (Figure 2). A decrease in frequency of naive B cells in the young group was detected when comparing controls to treated individuals (P < 0.05). As previously observed [15], the percentages of RM B cells in the non-treated and treated groups are significantly decreased as compared to controls in the middle (P < 0.01 and P < 0.05, respectively) and aged group (P < 0.05 and P < 0.01, respectively); the same clear trend was observed in the young group although the values did not reach statistical significance (Figure 2).

In addition, HIV-1 infection significantly increases the frequency of AM in both middle and aged groups (P < 0.01) and of TLM B cells in young, middle and aged groups (P < 0.01, P < 0.001 and P < 0.001, respectively) when comparing non-treated to the controls; the increased cell frequencies were corrected after ART administration in the aged and middle groups to levels comparable to the control group (Figure 2). Of interest is that an increased frequency of AM (P < 0.001) and TLM (P < 0.001) B cells was found in the treated young group when comparing to controls; this finding is likely due to the relatively short period of ART in these young individuals.

Regression analysis of the dependence of B cell subpopulation frequencies on age and treatment duration (Table 2) lends support for the positive effect of ART on correcting the levels of AM and TLM B cells in HIV-1 infected patients and further reinforces the conclusion that treatment and/or age have no effect on restoring the frequency of RM B cells in treated HIV-1 infected patients.

**Table 2:** **Regression analysis of the dependence of B cell subpopulation percentages on age and treatment duration**

**B cell subsets**	**Variables included in the model**			**R** ^**2**^	**P value**
	**Age**	**Treatment duration**	**Interaction**		
NAÏVE	+	+		0.179	**0.001**
Resting Memory				0.050	0.064
Active Memory		+		0.153	**0.001**
Tissue like Memory		+		0.083	**0.015**

Each row represents the regression result for one B cell subpopulation. For each population, the variables that were found predictive are marked with a “+”. R^2^ is the correlation coefficient between the specific B cell subpopulation and the chosen predictive variable(s). P values in bold indicate significant difference.

**Figure 3:**
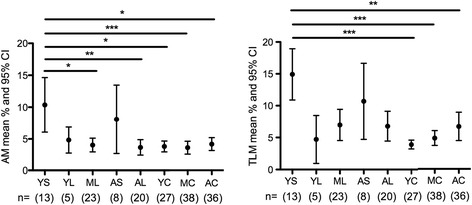
**Effect of treatment duration and age on AM and TLM subset frequencies.** To assess the effect of treatment and age on AM and TLM subset distribution we arbitrarily divided 69 of the 70 treated patients presented in Table 1 into 5 groups: 13 young with short treatment periods (YS) [age 21–30 and mean (range) length of ART treatment 1.8 (1–6) months], 5 young with long treatment periods (YL) [age 21–30 and 28.4 (18–48) months of ART], 23 in the middle age group with long treatment periods (ML) [age 31–50 and 66.8 (16–160) months of ART], 8 aged with short treatment periods (AS) [age 51–64 and 5.6 (2–12) months of ART], and 20 aged with long treatment periods (AL) [age 51–64 and 87 (15–163) months of ART]. We had only one patient in the middle group with a short treatment period and accordingly we excluded this patient from the analysis. The treated patients were compared to the control group divided into young (YC; N = 27), middle (MC; n = 38) and aged (AC; n = 36) according to Table 1. * = P < 0.05, ** = P < 0.01, *** = P < 0.001.

To assess the effect of treatment duration as well as age on frequencies of AM and TLM B cells, we subdivided our treated patients into groups with different age and ART duration, as illustrated in Figure 3. We compared the mean percentages of AM and TLM B cells of these treated, HIV-1 infected groups to the same age groups of the controls. We found that young HIV-1 infected patients with short ART duration have higher mean percentages of AM and TLM B cells significantly different from each of the control age groups (Figure 3). In addition the AM levels in the young HIV-1 infected patients with short ART duration are significantly different from the levels found in the treated patients included in the middle and aged group. These analyses confirm that the short treatment period in the young treated patients is associated with the increased frequencies of AM and TLM B cells; prolonged treatment appears to be effective in reducing the levels of AM and TLM B cells even in the young group of patients (Figure 3).

**Figure 4:**
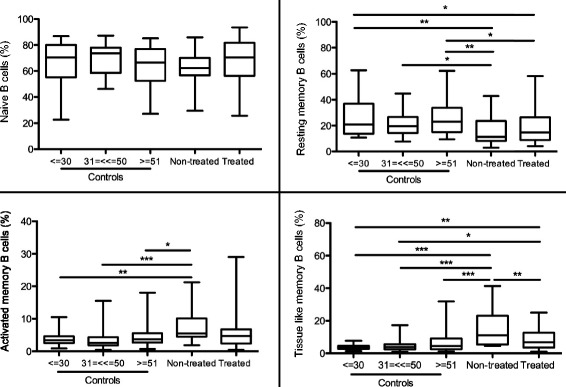
**B cell subsets from HIV-1 infected patients in relation to age groups from healthy controls.** B cell subsets from HIV-1 infected, non-treated and treated individuals of all ages were compared to the three age groups of healthy controls. The specimens included in the figure were obtained from the subjects presented in Table 1. The average percentages are illustrated for the different subsets of naive, resting memory (RM), activated memory (AM) and tissue-like memory (TLM) B cells. * = P < 0.05, ** = P < 0.01, *** = P < 0.001.

Finally, we compared each HIV-1 study population as a whole to the different age groups of the controls, in order to find whether the treated or non-treated group is more similar to a specific age group among the controls. We did not detect any significant difference between the groups in regard to the frequency of naive B cells (Figure 4). The non-treated patients have significantly higher percentages of AM (P < 0.01, P < 0.001 and P < 0.05, respectively) and TLM (P < 0.001) than all the age groups of the controls. However, the treatment decreased (P < 0.01 and P < 0.05, respectively) but did not correct the percentage of TLM B cells to what is found in the young and middle control groups. In contrast, the percentages of RM B cells in non-treated and treated group are lower than RM B cells of all control groups (Figure 4).

All in all, the above results show that while treatment corrects several of the effects of HIV-1 infection, neither treatment nor aging, nor their combination, affect the decrease in the percentage of RM B cells caused by HIV-1 infection.

### Expression of the activation markers CD25 and CD69 on B cell subpopulations

In order to understand whether phenotypic changes take place in B cell subsets with age in combination with HIV-1 infection, a panel of phenotypic markers including B cell inhibitory receptors PD1 and FcRL4 and activation molecules CD25 and CD69 was used. For the purpose we characterized specimens from young and aged healthy subjects (n = 30) and HIV-1 infected individuals (n = 63; 22 naïve and 41 on ART) (age ranges and characteristics indicated in Table 3).

**Table 3:** **Characteristics of young and aged individuals used for phenotypic characterization**

	**Controls**		**Treated**		**Non-treated**	
**Parameters**	**Young**	**Aged**	**Young**	**Aged**	**Young**	**Aged**
Number of patients	15	15	23	18	11	11
Age (mean age)	21-26 (23.9)	57-87 (62.7)	21-32 (26.9)	51-76 (58.2)	23-25 (24.3)	51-85 (63.9)
CD4 cells/μl (range)	ND	ND	645 (308–2010)	544 (314–1052)	637 (394–1096)	604 (336–1235)
Virus load copies/ml (range)	NA	NA	128 (0–893)	37.8 (0–343)	1.2×10^5^ (20-1×10^6^)	4.3×10^4^ (65–1.2×10^5^)
Length of ART Months (range)	NA	NA	14.4 (1–50)	20.1 (1–74)	NA	NA

ND = Not determined.

NA = Not applicable.

**Figure 5:**
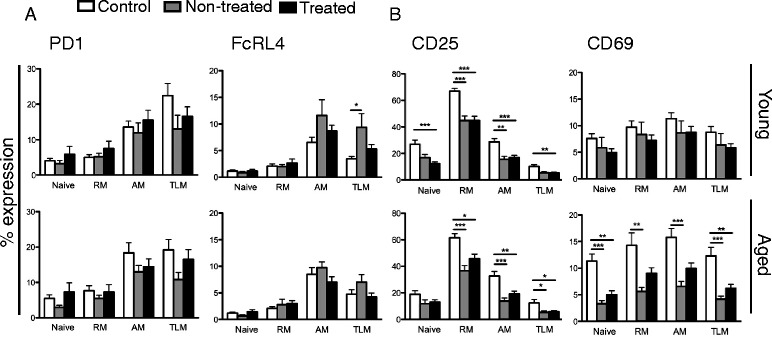
**Exhaustion and activation markers on B cell subsets.** Panel **A** shows the expression of surface markers of exhaustion (PD1 and FcRL4) and panel **B** markers of activation (CD25 and CD69) on B cell subsets which was compared in controls, non-treated and treated young and aged individuals. The specimens included in the figure were obtained from the subjects presented in Table 3. The average expression is illustrated for the different subsets of naive, resting memory (RM), activated memory (AM) and tissue-like memory (TLM) B cells. * = P < 0.05, ** = P < 0.01, *** = P < 0.001.

To evaluate the changes that HIV-1 infection induces in B cell phenotype, we first compared young individuals in the three study groups to each other in regard to the expression of inhibitory receptors on B cell subsets (Figure 5A). The expression of PD1 was not different among groups on any B cell subset studied. The expression of FcRL4 was significantly higher on TLM B cells in the non-treated group compared to the controls (P < 0.05). The comparison (Figure 5A) of the inhibitory receptor PD1 and FcRL4 on various B cell subsets of the three groups of aged individuals (control, non-treated, treated) revealed no difference in any B cell subset studied.

Interestingly, in young individuals, the expression of CD25 activation marker was significantly reduced on the RM (P < 0.001) and AM (P < 0.01) subsets of B cells from non-treated patients when compared to the controls (Figure 5B). Similarly, the expression of CD25 in the treated group was significantly lower on all B cell subsets (ranging from P < 0.001 to P < 0.01) when compared to the controls. HIV-1 infection did not alter the expression of CD69 on the B cell subsets of young infected individuals (Figure 5B).

As noticed for the young group of HIV-1 infected patients, the expression of CD25 in the aged group was significantly lower on RM, AM and TLM B cells from the non-treated when compared to the controls (P < 0.001; P < 0.001 and P < 0.05 respectively). Treatment did not restore any of these differences (Figure 5B). We also identified a lower expression of CD69 in the four B cell subsets Naive, RM, AM and TLM B cells of the aged non-treated patients (P < 0.001, P < 0.01, P < 0.001 and P < 0.001 respectively) when compared to the controls (Figure 5B). ART did not correct for the low expression of CD69 in B cells from HIV-1 infected individuals which remained low in all subpopulations and significantly differed for Naive (P < 0.01) and TLM B cells (P < 0.01).

When the levels of expression of CD25 and CD69 were compared in all groups of young and aged individuals (Additional file 3: Figure S3) the following observation were made: the level of CD25 on B cell subpopulations are equally low in young and old HIV-1 infected individuals and not corrected by ART treatment. Interestingly, the different CD69 expression detected in aged controls and HIV-1 infected patients appears to be the result of an increased expression of this molecule in aged healthy controls and does not reflect the virus effect on B cells from aged HIV-1 infected patients.

### The effect of age and length of therapy on B cell phenotypical changes

We next assessed whether differences in B cell phenotype of HIV-1 infected subjects on ART (including the low expression of CD69 and CD25) depend on age and length of therapy. For this purpose, we performed regression analysis including age, treatment duration and the interaction between them as continuous independent parameters, and assessed whether these parameters affect the phenotype of B cell subpopulations – which are the continuous dependent variables. A total of 41 HIV-1 infected subjects between 21–76 years of age, receiving ART were included in the analysis, 23 young and 18 aged (see Table 3 for virological and immunological details); the length of ART treatment, ranging between 1–74 months, was comparable in the two groups of young and aged treated patients.

Table 4 shows the results of the stepwise regression analysis. When analysing the phenotype patterns (including expression of PD1, FcRL4, CD25 and CD69) in all B cell subpopulations, we found only weak correlations between the specific subpopulations and the chosen predictive variable(s), as can be seen by the low R^2^ values. The only significant results (P <0.05) were seen in the AM and RM B cells expressing CD25, and, AM and TLM B cells expressing FcRL4. However, even for these significant values, the highest correlation coefficient (R^2^) was 0.172, indicating that only 17.2% of the total variation in the frequency of RM CD25 expressing B cells is explained by treatment duration. Our regression analysis (Table 4) shows that age, treatment duration and the interaction between them do not affect differences in the expression of exhaustion and activation markers on B cells from HIV-1 infected subjects undergoing treatment.

**Table 4:** **Regression analysis of B cell phenotypical changes in relation to age and treatment duration**

**B cell phenotype**	**Variables included in the model**			**R** ^**2**^	**P value**
	**Age**	**Treatment duration**	**Interaction**		
AM_PD1				0.033	0.258
RM_PD1				0.016	0.438
NAÏVE_PD1				0.002	0.771
TLM_PD1				0.003	0.737
AM_FcRL4			+	0.109	**0.035**
RM_FcRL4				0.020	0.372
NAÏVE_FcRL4				0.015	0.452
TLM_FcRL4		+		0.135	**0.018**
AM_CD25		+		0.127	**0.022**
RM_CD25		+		0.172	**0.007**
NAÏVE_CD25				0.045	0.185
TLM_CD25				0.017	0.423
AM_CD69				0.018	0.403
RM_CD69				0.030	0.276
NAÏVE_CD69				0.016	0.431
TLM_CD69				0.010	0.533

Each row represents the regression result for one B cell subpopulation. For each population, the variables that were found predictive are marked with a “+”. R^2^ is the correlation coefficient between the specific B cell subpopulation and the chosen predictive variable(s). P values in bold indicate significant difference.

### Expression of CD25 and CD69 on B cells correlates with the capacity of B cells to respond to in vitro activation stimuli by intracellular IL-6 expression

**Figure 6:**
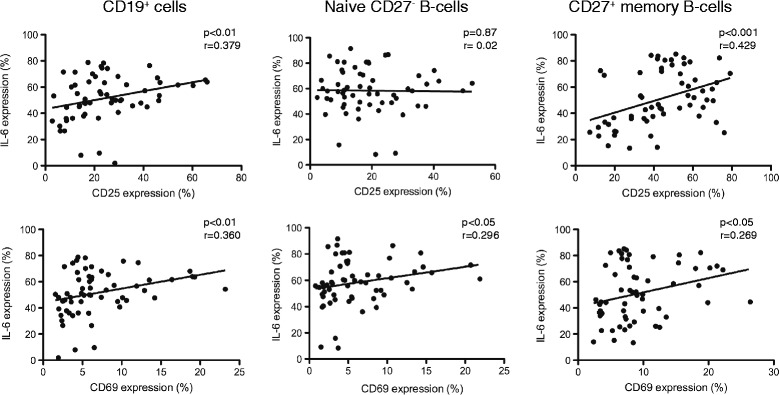
**Association of surface CD25 and CD69 expression on B cells with intracellular IL-6 expression.** The percentages of total, naïve and memory (CD27+) B cells expressing CD25 and CD69 were correlated with cells expressing intracellular IL-6 following in vitro stimulation with CpG and CD40L. Specimens from 60 individuals are included in the analysis: 21 HIV-1 infected individuals naïve to treatment, 19 HIV-1 infected patients on ART and 20 age matched controls. The P values were derived using the Spearman correlation test.

To determine if the low expression of surface CD25 and CD69 on B cells could be linked to any functional impairment in these cells, we stimulated cells from both controls and HIV-1 infected individuals and assessed the intracellular expression of the IL-6 cytokine (Figure 6). IL-6, a multifunctional cytokine, important for many aspects of B cell differentiation and antibody production is produced by multiple cell types including activated B cells. A significant positive correlation was found between the surface CD25 expression and percentages of total B cells (P < 0.01 and r = 0.379) and CD27+ memory B cells (P < 0.001 and r = 0.429) expressing IL-6 following in vitro stimulation (Figure 6). A similar positive correlation was found between IL-6 expression and surface CD69 expression on all B cells (P < 0.01 and r = 0.360), naive B cells (P < 0.001 and r = 0.450) and memory B cells (P < 0.05 and r = 0.269) (Figure 6). These results indicate that low surface expression of activation markers CD25 and CD69 on the B cell is linked to a weaker response of B cells to exogenous activating stimuli.

## Discussion

HIV-1 infection, in view of its chronicity, has been postulated to induce detectable changes of premature aging in the immune system. The changes occurring in B cells during aging of HIV-1 infected individuals have yet not been fully characterized. In the current study, to investigate the impact of HIV-1 infection on B cell aging, we have compared the frequency, surface markers and intracellular IL-6 expression in response to activation of multiple B cell subsets in the blood of healthy and HIV-1 infected individuals.

It is still controversial whether age affects the total number of B cells, as opposite results have been presented in this context [25,26]. When subdivided into subsets, a decline of naïve B cells has been shown during aging in healthy individuals, whereas memory B cells (CD27^+^) have been found slightly decreased in some reports and increased in other studies [27-29]. Memory B cells against specific pathogens, however, increase during aging as demonstrated for cytomegalovirus (CMV) [30]. In our control group, the levels of naïve B cells and all memory B cell populations (RM, AM and TLM B cells) remained constant at different ages.

Interestingly, we found that HIV-1 infection impacts similarly on the distribution of B cell subpopulations in all age groups, but the effects of treatment are not the same. In our study, depletion of RM B cells taking place in all age groups during HIV-1 infection was not corrected by ART. HIV-1 infected patients have increased percentages of AM and TLM B cells in the middle and aged groups, but the treatment, when provided for more than 12 months, significantly reduces these percentages to levels that are comparable to those of controls (Figure 3); longer treatment periods in the young HIV-1 infected group also appears to normalize the levels of AM and TLM B cells (Figure 3). Thus, the restored frequencies of AM and TLM B cells in the middle and aged group are likely the result of longer treatment periods, allowing time for ART to correct for alteration to the B cell phenotype, rather than age itself.

Furthermore, our data show important differences in the surface expression of B cell markers between healthy individuals and subjects with HIV-1 infection. Both aged and young controls had the highest expression of CD25 on all B cell subsets when compared to HIV-1 infected individuals (Additional file 3: Figure S3). CD25 (alpha chain of the IL-2 receptor), although present on some naive B cells, is highly expressed on the surface of CD27^+^ memory B cells [31]. Low CD25 expression has been previously detected on memory B cells of HIV-1 patients [32]. In the same study the levels of plasma IL-2 were directly correlated with the low frequency and survival of memory B cells in a STAT5 and Foxo3a dependent manner [32]. Serum levels of IL-2 are decreased during HIV-1 infection [33] and decreased production of IL-2 by T-cells, the main IL-2 producing cells, has been shown in healthy individuals with advanced age [34]. Co-cultures of CD25 expressing B cells with CD4^+^ T-cells in the presence of low IL-2 concentrations resulted in T-cell anergy, while high IL-2 concentration induced T-cell proliferation [35]. It has also been shown that CD25 expressing B cells induce FoxP3^+^ regulatory T-cells in co-culture [36]. The low expression of CD25 on memory B cells during HIV-1 infection may thus lead to two important consequences: 1) impaired proliferation and survival of B cells, and 2) impaired interactions with T-cells and T-cell differentiation.

Chronic activation during HIV-1 infection results in the accumulation of exhausted B cell subsets expressing multiple inhibitory receptors such as FcRL4 and PD1, which contribute to inefficient immune responses against the virus. Expression of PD1 on B cells has been linked with memory B cell depletion and impaired humoral responses in SIV-infected macaques [37]. Additionally, PD1 has been shown to be present on multiple B cell subsets in healthy individuals and has an inhibitory effect on B cell activation [38]. FcRL4 or Fc receptor–like proteins are trans-membrane proteins previously described to be expressed on a unique population of memory B cells, termed TLM, in human lymphoid tissues near epithelial surfaces [39]. We could not detect differences in PD1 expression for the different subsets; the expression of FcRL4 was however increased in TLM B cells of young non-treated patients and this change was corrected by ART. Previously it has been shown that silencing of the FcRL4 receptor with siRNA in TLM B cells from HIV-1 patients resulted in increased secretion levels of HIV-1 specific antibody, in addition to production of B cell associated cytokines and chemokines [40].

The expression of FcRL4 on human B cell lines disrupts immune synapse formation and blocks antigen induced BCR signaling, in addition to CD69 expression [41]. CD69 is an early activation marker mainly expressed by activated T, B and natural killer cells. CD69 acts down-stream the IFN alpha/beta signaling and affects lymphocyte migration from lymph nodes through S1P_1_ inhibition [42]. When comparing young and aged control individuals, there is a higher expression of CD69 in aged control individuals on all the B cell subsets studied (for naïve and TLM B cells P < 0.05). The same elevation in CD69 expression could not be measured on B cells of aged HIV-1 infected individuals (Figure 5 and Additional file 3: Figure S3). Whether elevation of CD69 expression in the aged controls, but not in the patients, play a relevant biological role for B cell immunology needs to be verified in additional studies.

We could further show that B cells expressing low levels of CD25 and CD69, including naive and memory B cells and independently of the studied groups, have a poor response to exogenous stimuli as detected by the low expression of intracellular IL-6. IL-6 a pro-inflammatory, multifunctional cytokine plays a key role in B cells activation, terminal differentiation and antibody production [43]. High serum levels of IL-6 during HIV-1 infection have been previously associated with increased levels of activated memory B cells [44]. Further studies using larger number of HIV-1 infected patients need to be conducted to clarify the link between B cell phenotype and B cell activation.

It can then be asked whether aging results in profound changes in the phenotype and function of B cell subsets during HIV-1 infection. The decline of RM B cells, although due to virus infection rather than to the aging process, affects dramatically all age groups during HIV-1 infection and is not reverted by ART treatment. In addition, B cell subpopulations of HIV-1 infected patients of all age have reduced levels of the CD25 marker (alpha chain of the IL-2 receptor) which is not corrected by ART.

## Conclusions

The present study shows that RM B cells in young and aged HIV-1 infected patients are reduced in frequency and exhibit a reduced expression level of the activation marker CD25. On the other hand, elevated frequencies of AM and TLM B cells in young and aged patients, are correct by prolonged ART treatment. Age per se does not appear to worsen impairment induced in the B cell compartment by HIV-1 infection.

These pathological findings in HIV-1 infected patients suggest that, in spite of prolonged treatment, some aspects of B cell impairments persist in both young and aged HIV-1 infected patients. In view of these findings, following prolonged ART, re-vaccination against common pathogens may need to be taken into account for this particular population.

## Methods

### Study population

Subjects included in the study were HIV-1 infected individuals naive to treatment [n = 35; defined as non-treated; mean age (age range) 41.3 (23–85) years], on ART [n = 70; defined as treated; 43.9 (21–76) years], and HIV-1 negative healthy individuals [n = 101; defined as controls; 42.9 (21–87) years]. The mean CD4^+^ T-cell count/μl in the non-treated group was 601 (range 278–1357) and in the treated group 591 (222–2010). The viral load ranged between 20-1×10^6^ copies/μl in the blood of non-treated patients and 0–893 copies/μl in treated patients. The individuals included in the study were divided into 3 age group categories according to the overall population mean age: Low: Age < Mean - 1 SD, Medial: (Mean-1 SD < Age < Mean + 1 SD) and High: Age > Mean + 1 SD.

In order to elucidate the impact of age and HIV-1 infection on phenotypic markers of B cell subpopulations, a panel of specimens from individuals belonging to the three groups defined above, a total of 30 healthy subjects and 63 HIV-1 infected individuals (22 naïve to treatment and 41 treated) was further characterized for surface phenotype. We first identified the youngest and the oldest individuals in our 2 groups of HIV-1 infected subjects and then matched the healthy controls to the patients. The age, CD4^+^ T-cell counts, viral load and ART duration for these individuals are presented in Table 3. The young and aged groups of HIV-1 treated patients were matched for length of treatment (up to 74 months).

For the regression analysis presented in Table 4 a total of 41 HIV-1 infected subjects between 21–76 years of age, receiving ART were included in the analysis, 23 young and 18 aged (see Table 3 for virological and immunological details). The length of ART treatment was comparable in the two groups of young and aged treated patients.

### Cell isolation and phenotyping

Peripheral blood mononuclear cells (PBMCs) were isolated using Ficoll gradient centrifugation (Lymphoprep, Axis-Shield Poc AS, Oslo, Norway). Cells were washed, counted using Countess automated cell counter (Invitrogen, NY, USA) and used for flow cytometry analyses. The study was approved by the ethical committee at Karolinska Institutet and informed consent was obtained from all the individuals included in the study.

For flow cytometric analyses, isolated PBMCs were plated at 1-2×10^6^ cells per sample and stained using fluorochrome-conjugated antibodies in different combinations: V450 CD3 (clone UCHT1), PE CD4 (clone RPA-T4), V500 CD8 (RPA-T8), PerCp-Cy5.5 anti-CD19 (clone HIB19), FITC anti-CD10 (W8E7), V450 anti-CD27 (MT271) and anti-CD21 (B-ly4), APC anti-CD21 (B-ly4), PE anti-CD27 (M-T271), anti-PD1 (MIH4), anti-CD25 (M-A251) and anti-CD69 (FN50) all from (BD Biosciences, CA, USA). APC anti-FcRL4 (413D12) was from BioLegend. LIVE/DEAD Fixable Near-IR kit (Invitrogen) was used to exclude the dead cells from analyses. Cells were washed three times before being fixed in 1% formaldehyde. All antibodies were used in the concentrations determined by titration experiments. Matched isotype controls or FMO were used to set up the gates. Fluorescence intensities were measured with Cyan ADP (Beckman Coulter) and data were analysed using FlowJo, version 9.4.11 (Tree star, OR, USA). In order to derive the CD4:CD8 ratio for T cells from the healthy controls, the frozen PBMCs were used.

### Identification of B cell subsets

Peripheral whole B cell population was gated out as CD19^+^ cells after exclusion of dead cells. Whole B cells were further subdivided into various B cell subsets: Naive CD19^+^CD10^**−**^CD21^+^CD27^**−**^, Activated Memory (AM) CD19^+^CD10^**−**^CD21^**−**^CD27^+^, Resting Memory (RM) CD19^+^CD10^**−**^CD21^+^CD27^+^ and Tissue Like Memory (TLM) CD19^+^CD10^**−**^CD21^**−**^CD27^**−**^ B cells; the gating strategy is illustrated in Additional file 1: Figure S1. Whole memory B cells were identified as CD19^+^CD10^−^CD27^+^.

### Intracellular cytokine detection

PBMCs were cultured at a concentration of 2×10^6^ cells/ml in RPMI-1640 containing L-glutamine, 10% FCS and antibiotics in medium only or stimulated with 10 μg/ml CpG-B (InvivoGen, California, USA) and 1 μg/ml CD40L (InvivoGen) for 48 h. For the last 5 hours of the culture, an additional stimulation with 50 ng/ml PMA and 1 μg/ml Inomycin (Sigma-Aldrich, MO, USA) in the presence of Golgistop used at 1:1000 dilution (BD) was performed. Cells were first stained for cell surface markers PerCp anti-CD19, FITC anti-CD10 and V450 anti-CD27 in addition to LIVE/DEAD staining. In next step cells were fixed and permeabilized using BD Cytofix/Cytoperm kit and stained with PE anti-IL-6 (MQ2-6A3) and matching isotype control, both from (BD). Cells were washed and fixed in 1% formaldehyde before the analysis were conducted using a Cyan flow cytometer. Collected data were analysed using FlowJo, version 9.4.11 (Tree star).

### Statistical analysis

One-way ANOVA with Newman-Keuls post-test was used when multiple groups were compared. The correlation data were analysed using the Spearman correlation test.

In order to dissect the effects of age versus length of therapy on phenotypical alterations (expression of PD1, CD25, CD69 and FcRL4) taking place on B cell subpopulations, we performed regression analysis. We used a stepwise regression method, which uses an automatic procedure in order to decide which of the independent variables are the predictive ones. Age, treatment duration and the interaction between them are the possible predictive variables, and they were included in the regression analysis according to the p value of the F-tests. We used the backward elimination method for choosing the predictive variables. This method starts with all possible predictive variables as candidates, deletes each variable, one by one, and tests whether the deletion improved the correlation between the B cell phenotype and the predictive variables that were not deleted. This process is repeated until no further improvement in the correlation is achieved.

Data were analysed using SPSS version 20 and graphed using Prism version 5.0a (GraphPad Software, La Jolla, California, USA).

## Additional files

Additional file 1: Figure S1. Gating strategy for identification of B cell subsets. Additional file 2: Figure S2. Percentages of B cell subpopulations in control individuals, non-treated and treated HIV-1 patients. The specimens included in the figure were obtained from 101 healthy controls, 35 HIV-1 infected subjects naïve to treatment and 70 HIV-1 infected subjects receiving ART (Table 1). * = P < 0.05, ** = P < 0.01, *** = P < 0.001. Additional file 3: Figure S3. Changes in expression of phenotype markers CD25 and CD69 in relation to age and infection. The expression of phenotype markers was compared in all subsets of B-cells between young controls and aged controls, HIV-1 infected individuals naive to treatment and on treatment. * = P < 0.05, ** = P < 0.01, *** = P < 0.001.

## Abbreviations

HIV-1: Human immunodeficiency virus-type 1
ART: Antiretroviral therapy
PBMCs: Peripheral blood mononuclear cells
RM B cells: Resting memory B cells
AM B cells: Activated memory B cells
TLM B cells: Tissue like memory B cells
